# Anti-Inflammatory Chemical Profiling of the Australian Rainforest Tree *Alphitonia petriei* (Rhamnaceae)

**DOI:** 10.3390/molecules21111521

**Published:** 2016-11-11

**Authors:** Ritesh Raju, Dhanushka Gunawardena, Most Afia Ahktar, Mitchell Low, Paul Reddell, Gerald Münch

**Affiliations:** 1 School of Medicine, Western Sydney University, Locked Bag 1797, Penrith, NSW 2751, Australia; r.raju@westernsydney.edu.au (R.R.); d.gunawardena@westernsydney.edu.au (D.G.); m.ahktar@uws.edu.au (M.A.A.); 2 Ecobiotics Limited, 7 Penda Street, Yungaburra, QLD 4884, Australia; Paul.Reddell@ecobiotics.com.au; 3 Molecular Medicine Research Group, Western Sydney University, Locked Bag 1797, Penrith, NSW 2751, Australia; 4 National Institute of Complementary Medicine (NICM), Western Sydney University, Locked Bag 1797, Penrith, NSW 2751, Australia; mitchell.low@westernsydney.edu.au

**Keywords:** anti-inflammatory, alphitolic acid, nitric oxide, TNF-α, natural products

## Abstract

Chronic inflammation is an important pathological condition in many human diseases, and due to the side effects of the currently used non-steroidal anti-inflammatory drugs, discovery of novel anti-inflammatory drugs is of general interest. Anti-inflammatory activity guided compound isolation from the plant *Alphitonia petriei* led to the isolation of the known plant sterols emmolic acid (**1**), alphitolic acid (**2**), *trans-* and *cis*-coumaroyl esters of alphitolic acid (**3** and **4**) and betulinic acid (**5**). A detailed spectroscopic analysis led to the structure elucidation of the alphitolic acid derivatives (**1**–**5**), and the semi-synthetic emmolic acid acetate (**6**). When tested in LPS (Lipopolysaccharides) + IFN-γ (Interferon gamma) activated RAW 264.7 macrophages, all compounds except (**1**) exhibited potent anti-inflammatory activity (IC_50_ values as low as 1.7 μM) in terms of downregulation of NO and TNF-α production, but also demonstrated some considerable cytotoxicity.

## 1. Introduction

Chronic inflammation is an important etiological condition for various peripheral chronic diseases, including allergies, asthma, rheumatoid arthritis, atherosclerosis, Celiac and Crohn’s disease, metabolic syndrome/diabetes, periodontitis and psoriasis [1,2,3,4]. Furthermore, increasing evidence suggests that systemic low grade inflammation is a contributing factor in age-related neurodegenerative diseases, such as multiple sclerosis, traumatic brain injury, Alzheimer’s disease and Parkinson’s disease [5,6,7,8]. Inflammatory cells, including macrophages and microglia are considered to play a major role in the body’s response to immunogenic challenges, producing large amounts of superoxide (converted to hydrogen peroxide), nitric oxide (NO) and pro-inflammatory cytokines such as tumor necrosis factor alpha (TNF-α) [9], that aggravate and propagate inflammation, disrupt the normal function of cells and even may cause cell death [10,11]. For example, the pro-inflammatory cytokine, TNF-α, binds to two tumour necrosis factor receptors TNFR1 and TNFR2 and, depending on the adaptor protein, activates various signalling pathways including NF-κB, p38 and JNK which can lead to proliferation, cell migration, apoptosis and necrosis [12]. NO is a signalling molecule with diverse cellular roles that is also released by macrophages for defence [13]. The toxicity of NO is attributed to its ability to bind to proteins that contain heme, iron, copper or organic side groups, such as thiols, resulting in protein modification and the shutting-down of cellular activities [14]. Therefore, limiting inflammatory cytokine and NO production by activated macrophages/microglia should be beneficial for prevention of systemic and local inflammation and is potentially cytoprotective. 

To date, pharmacotherapy of inflammatory conditions is based on the use of non-steroidal anti-inflammatory drugs (NSAIDs). However, NSAIDs can cause serious gastrointestinal toxicity such as gastric bleeding and the formation of stomach ulcers. Some NSAIDs, particularly COX-2 inhibitors, have been linked to increased blood pressure, greatly increased risk of congestive heart failure, thrombosis and myocardial infraction [15]. Thus, the identification of anti-inflammatory treatments with fewer adverse effects is an important task. 

As part of a program to identify novel anti-inflammatory compounds from Australian rainforest plants, we have screened a variety of extracts. One of the most potent extracts was derived from the leaves of *Alphitonia petriei. Alphitonia petriei* (Braid and C.T.White (family RHAMNACEAE)) is a common rainforest tree in eastern Australia occurring from northern New South Wales to the Torres Strait Islands in Queensland. Previous phytochemical studies of this genus have yielded ceanothic, betulinic, and alphitolic acids, and the novel triterpene, aphitexolide from the bark of *A. excelsa* [16]. More recently, the chloroform extract of the bark of *Alphitonia petriei* (collected from Paluma Range in north Queensland) led to the discovery of 2-ketobetulinic acid and betulinic acid, both of which showed antibacterial activity against *Bacillus subtilis* [17].

**Figure 1:**
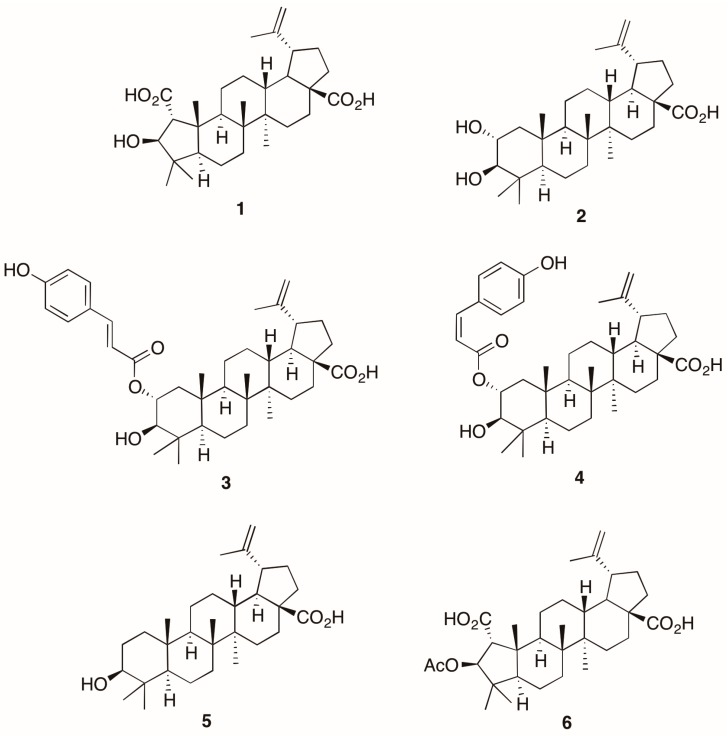
Chemical structures of **1**–**6**.

In this study, we isolated six natural and semi-synthetic plant sterols (**1**–**6**) (Figure 1) from *A. petriei.* A detailed account on the isolation, characterisation and anti-inflammatory bioactivity profiling of the sterols is described herein.

## 2. Results and Discussion

The raw plant material consisting of the stem and leaves of *Alphitonia petriei* (250 g) was extracted twice in EtOH, and dried in vacuo, giving a crude extract of 48.7 g. This crude extract was resuspended in MeOH and purified through reversed phase semi-prep HPLC (PhenylHexyl column, 250 × 9.4 mm, Phenomenex).

Detection of five plant sterols, emmolic acid (**1**), alphitolic acid (**2**), *trans*-coumaroyl alphitolic acid (**3**), *cis*-coumaroyl alphitolic acid (**4**), betulinic acid (**5**) and the semi-synthetic derivative, emmolic acid acetate (**6**) was performed.

Detailed NMR spectroscopy interpretation of each of the compounds, led us to characterize and document their anti-inflammatory activity, initially identified from the plant ethanolic extract. 

Compounds **1**–**6** (Figure 1) were screened for their anti-inflammatory activity. The bioactivities of compounds against NO and TNF-α production induced by LPS + IFN-γ in RAW 264.7 cells were examined using the Griess assay (for NO, *n* = 8) and TNF-α (by ELISA, *n* = 3). In addition to the natural products isolated, we then synthesized the acetate of (**1**) [18], and found that emmolic acid acetate [19] (**6**) rendered the molecule from an inactive state to having moderate anti-inflammatory activity (Table 1), which has not been documented before for emmolic acid [19]. Compounds **3** [20], **4** [21] and **5** [22] displayed potent NO inhibition with IC_50_ values in the range of 1–8 μM, which is orders of magnitude higher than the conventional NSAIDs. Compound **4** also showed strong inhibition of TNF-α. In terms of structure–activity relationships, it is interesting to note that the attachment of a coumaroyl oxy moiety on alphitolic acid moiety (compound **3**) increased the potency of NO inhibition by ten-fold when compared with compound **2** [23].

Jeong et al. reported the isolation of alphitolic acid and betulinic acid from *Callistemon lanceolatus*, and assayed them with respect to the inhibitory effects of NO production in LPS-stimulated RAW 264.7 cells, but did not provide IC_50_ values for these compounds [24]. Interestingly, they described betulinic acid 3-*O*-caffeate (containing a 3,4-dihydroxyhydrocinnamic acid attached to the sterol scaffold at position 3) as their most active compound in term of NO inhibition [24]. It agrees with our finding that our compound **3** with the additional trans-4-hydroxycinnamic (*p*-coumaric) acid linked to the A sterol ring increases the anti-inflammatory potency of the plant sterol quite significantly. It has to be noted that the IC_50_ values between their study and our study cannot be directly compared, as the studies did not use the same pro-inflammatory activators (LPS vs. LPS + IFN-γ).

Very few other studies have reported anti-inflammatory activities of our isolated compounds in vivo. For example, alphitolic acid was shown to inhibit 12-*O*-tetradecanoylphorbol 13 acetate (TPA)-induced topical inflammation in mice ears [25] (all except compound **1** showed some degree of cell death, and the therapeutic index (LD_50_/ED_50_) was not more than 3.5 for the best compounds). These data suggest that compounds (**2**–**6**) need to be tested in primary cells to establish if their toxicity is only directed against cancer cells. Only if they are less toxic to normal cells than to cancer cells, can they be evaluated as potential antitumor agent as well. Alphitolic acid (ALA) (compound **2**), has already been investigated in this regard by Bai et al. They have shown that it suppresses the proliferation of SCC4 and SCC2095 OSCC cells with IC_50_ values of 12 and 15 μM, respectively, via apoptotic induction, which is close to our value of 18.6 µM. Mechanistically, ALA blocked Akt–NF-κB signalling, which might be related to its anti-inflammatory mechanisms. Moreover, Bai et al. have shown that ALA induced autophagy, as evidenced by increased expression of the autophagy biomarkers Beclin 1, Atg7, and LC3B-II, and autophagosome formation. ALA increased p53 phosphorylation/expression, accompanied by parallel decreases in the expression of the oncogenic E3 ligase MDM2, and shRNA-mediated knockdown of p53 partially rescued ALA-mediated cytotoxicity [26].

In conclusion, we have isolated and documented the inhibition of LPS and IFN-γ induced NO, TNF-α production and also reported the cytotoxicity of the plant sterols **1**–**6** from the genus *Alphitonia* including their respective IC_50_ values.

## 3. Method and Materials

### 3.1. General Information

Bovine serum albumin, lipopolysaccharide (LPS) (Salmonella serotype), *N*-(1-1-napthyl) ethylenediamine dihydrochloride, resazurin sodium 10%, streptomycin, sulfanilamide, tetra methyl benzidine (TMB), ibuprofen, diclofenac sodium and Trypan blue were purchased from Sigma-Aldrich (Castle Hill, Australia). Dulbecco’s modified Eagle’s medium (DMEM), Phosphate buffer saline (PBS) foetal bovine serum (FBS) and glutamine were GIBCO brands purchased from Life Technologies (Mulgrave, Australia). Recombinant IFN-γ and TNF-α ELISA kits were purchased from PeproTech Asia (Rehovot, Israel). Ninety-six-well cell culture plates were initially from Greiner Bio-One, but were later replaced by Eppendorff plates as they show improved evaporation characteristics (no edge effect).

NMR spectra were recorded on a Bruker Ascend 400 MHz spectrometer (Bruker Biospin GmbH, Bremen, Germany), in the solvents indicated and referenced to residual ^1^H signals in deuterated solvents. HRMS was carried out using a Waters Xevo Q-TOF mass spectrometer operating in the positive ESI mode.

### 3.2. Compound Isolation and Semisynthesis of ***6***

HPLC was carried out using an Agilent 1290 series (Santa Clara, CA, USA). Leaves and stems were collected from three mature *Alphitonia petriei* trees growing in a basalt-derived soil on the edge of complex notophyll vine forest on the Evelyn Tablelands of north Queensland (17°29.0′ S, 145°29.5′ E, altitude 1090 m). All of the plant material was frozen within 24 h of collection and was stored in a freezer required for extraction. The leaves and bark of *A. petriei* (250 g) were ground up into a fine powder and extracted in ethanol (500 mL × 2) to yield a crude extract of 48.7 g. This crude was resuspended in MeOH and subjected directly through reversed phase semi-prep HPLC (PhenylHexyl column, 250 × 9.4 mm, Phenomenex) eluting with a gradient starting from 10% MeCN/H_2_O to a 100% MeCN (with a constant 0.01% TFA modifier) over 30 min and held at 100% MeCN for 10 min to yield **1** (t_R_ = 19.8 min, 2.0 mg), **2** (t_R_ = 27 min, 2.1 mg), **3** (t_R_ = 31.5 min, 1.1 mg), **4** (t_R_ = 32.5 min, 0.6 mg), and **5** (t_R_ = 33.6 min, 3.6 mg).

#### Compound **6** (Emmolic Acid Acetate)

A solution of **1** (2.0 mg) in pyridine (300 µL) and acetic anhydride (300 µL) was stirred overnight, after which the reaction was concentrated to dryness and resuspended in MeOH and purified by HPLC (Jupiter C_18_ column, 250 × 4.6 mm, 5 µm, 1 mL·min^−1^, gradient from 10% to 100% MeCN over 20 min, with a hold at 100% MeCN for 5 min) to yield the emmolic acid acetate (**6**) (t_R_ = 20.6 min, 1.2 mg, 60%) as a white amorphous powder.

### 3.3. Cell Lines and Cell Culture

#### 3.3.1. Maintenance of RAW 264.7 Macrophages

RAW 264.7 macrophages were grown in 175 cm^2^ flasks on DMEM containing 5% foetal bovine serum (FBS) that was supplemented with penicillin (100 μg/mL), streptomycin (100 μg/mL) and l-glutamine (2 mM). The cell line was maintained in 5% CO_2_ at 37 °C, with media being replaced every 3–4 days. Once cells had grown to confluence in the culture flask, they were removed using a rubber policeman, as opposed to using trypsin, which can remove membrane-bound receptors [16]. The cell suspension was concentrated by centrifugation for 3 min at 900 rpm and re-suspended in a small volume of fresh DMEM (with 1% antibiotics and 5% FBS); cell densities were estimated using a Neubauer counting chamber. The cell concentration was adjusted with DMEM (with 1% antibiotics and 5% FBS) to obtain 60,000 cells/100 μL cell suspension. An aliquot of 100 μL of this cell suspension was dispensed into the wells of 96-well plates. Plates were incubated at 37 °C, 5% CO_2_ for 18 h before the activation experiments were carried out.

#### 3.3.2. Activation of RAW 264.7 Macrophages

For assays, 90 μL of each concentration of compounds (8 concentrations made by serial dilution from 0 µg/mL to 36 µg/mL compound in DMEM) were added an hour prior to addition of 10 µL of activator. A combination of 10 μg·mL^−1^ LPS and 10 U·mL^−1^ (1 unit = 0.1 ng/mL) IFN-γ, were used for activation. After activation, the cells were incubated for 24 h at 37 °C and then NO, TNF-α and cell viability was determined. Unactivated cells (exposed to media alone) were used as negative control and activated cells as positive control.

#### 3.3.3. Determination of Nitrite (as a Measure of Nitric Oxide Production) by the Griess Assay

Nitric oxide was determined by Griess reagent quantification of nitrite. Griess reagent was freshly made up of equal volumes of 1% sulfanilamide and 0.1% napthyethylene-diamine in 5% HCl. From each well, 50 µL of supernatant was transferred to a fresh 96-well plate and mixed with 50 µL of Griess reagent and measured at 540 nm in a POLARstar Omega microplate reader (BMG Labtech, Mornington, Australia). The concentration of nitrite was calculated using a standard curve with sodium nitrate (0–500 μM), and linear regression analysis.

#### 3.3.4. Determination of TNF-α by ELISA

The diluted supernatants were used for determination of TNF-α using a commercial sandwich ELISA (Peprotech) according to the manufacturer’s protocol. In brief, the capture antibody was used at a concentration of 1.5 μg·mL^−1^ in PBS (1.9 mM NaH_2_PO_4_, 8.1 mM Na_2_HPO_4_, 154 mM NaCl) (pH 7.4). Serial dilutions of TNF-α standard from 0 to 10,000 pg·mL^−1^ in diluent (0.05% Tween-20, 0.1% BSA in PBS) were used as internal standard. TNF-α was detected with a biotinylated second antibody and an Avidin peroxidase conjugate with TMB as detection reagent. The colour development was monitored at 655 nm, taking readings every 5 min. After approximately 30 min the reaction was stopped using 0.5 M sulphuric acid and the absorbance was measured at 450 nm using a POLARstar Omega microplate reader (BMG Labtech, Mornington, Australia), expressed as a percentage in control cells after conversion of the concentrations, using a standard curve constructed with defined concentrations of TNF-α. Curve fitting of this standard curve and extrapolation of experimental data were performed using non-linear regression analysis.

#### 3.3.5. Determination of Cell Viability by the Alamar Blue Assay

One hundred microlitres of Alamar Blue solution (10% Alamar Blue (Resazurin) in DMEM media) was added to each well, incubated at 37 °C for 2 h. After incubation, fluorescence intensity was measured with the microplate reader (excitation at 530 nm and emission at 590 nm) and results were expressed as a percentage of the intensity of that in control cells, after background fluorescence was subtracted.

### 3.4. Statistical Analysis

Data calculations were performed using MS-Excel 2010 software. IC_50_ values were obtained by using the sigmoidal dose–response function in GraphPad Prism. The results were expressed as mean ± standard deviation (SD).

## Figures and Tables

**Table 1:** Inhibition of LPS + IFN-γ induced nitric oxide and TNF-α (Tumor Necrosis Factor-alpha) production as well as cytotoxicity of compounds **1**–**6** in comparison to classical NSAIDs (statistical analysis is provided in Supplementary Materials Table S7).

Compound	NO Inhibition (IC_50_ in μM) (*n* = 8)	TNF-α Inhibition (IC_50_ in µM) (*n* = 3) (in μM) (IC_50_ in μM)	Cytotoxicity (LC_50_ in μM) (*n* = 8)	Therapeutic Index (TI) (Compared to NO)
**1**	>36	>36	>36	
**2**	17.6 ± 0.7	22.7 ± 1.1	18.6 ± 0.8	1
**3**	1.7 ± 0.3	10.9 ± 2.3	4.8 ± 0.9	2.8
**4**	3.5 ± 0.5	5.6 ± 0.4	8.0 ± 0.3	2.3
**5**	8.3 ± 1.0	23.5 ± 3.7	29.1 ± 4.4	3.5
**6**	14.7 ± 0.6	>36	13.1 ± 2.5	0.9
NSAID				
Diclofenac	217 ± 43	>333	>333	
Ibuprofen	1155 ± 133	723 ± 261	>1500	

## Supplementary Materials

Supplementary materials can be accessed at: http://www.mdpi.com/1420-3049/21/11/1521/s1.

## References

[1] Libby P., Ridker P.M., Maseri A. (2002). Inflammation and atherosclerosis. Circulation.

[2] Panettieri R.A. (2002). Airway smooth muscle: An immunomodulatory cell. J. Allergy Clin. Immunol..

[3] Goronzy J.J., Weyand C.M. (2005). Rheumatoid arthritis. Immunol. Rev..

[4] Kesmarky G., Feher G., Koltai K., Horvath B., Toth K. (2006). Viscosity, hemostasis and inflammation in atherosclerotic heart diseases. Clin. Hemorheol. Microcirc..

[5] Eikelenboom P., Bate C., Gool W.A.V., Hoozemans J.J.M., Rozemuller J.M., Veerhuis R., Williams A. (2002). Neuroinflammation in Alzheimer’s disease and prion disease. Glia.

[6] Venigalla M., Sonego S., Gyengesi E., Sharman M.J., Münch G. (2015). Novel promising therapeutics against chronic neuroinflammation and neurodegeneration in Alzheimer’s disease. Neurochem. Int..

[7] Varley J., Brooks D.J., Edison P. (2014). Imaging neuroinflammation in Alzheimer’s and other dementias: Recent advances and future directions. Alzheimer’s Dement..

[8] Durrenberger P.F., Fernando F.S., Kashefi S.N., Bonnert T.P., Seilhean D., Nait-Oumesmar B., Schmitt A., Gebicke-Haerter P.J., Falkai P., Grunblatt E. (2014). Common mechanisms in neurodegeneration and neuroinflammation: A BrainNet Europe gene expression microarray study. J. Neural Transm..

[9] Meiners I., Hauschildt S., Nieber K., Münch G. (2004). Pentoxyphylline and propentophylline are inhibitors of TNF-alpha release in monocytes activated by advanced glycation endproducts. J. Neural Transm..

[10] Münch G., Gasic-Milenkovic J., Dukic-Stefanovic S., Kuhla B., Heinrich K., Riederer P., Huttunen H.J., Founds H., Sajithlal G. (2003). Microglial activation induces cell death, inhibits neurite outgrowth and causes neurite retraction of differentiated neuroblastoma cells. Exp. Brain Res..

[11] Holmquist L., Stuchbury G., Steele M., Münch G. (2007). Hydrogen peroxide is a true first messenger. J. Neural Transm. Suppl..

[12] McCoy M.K., Tansey M.G. (2008). TNF signaling inhibition in the CNS: Implications for normal brain function and neurodegenerative disease. J. Neuroinflam..

[13] Ohshima H., Tsuda M., Adachi H., Ogura T., Sugimura T., Esumi H. (1991). l-arginine-dependent formation of N-nitrosamines by the cytosol of macrophages activated with lipopolysaccharide and interferon-gamma. Carcinogenesis.

[14] Stamler J.S., Singel D.J., Loscalzo J. (1992). Biochemistry of nitric oxide and its redox-activated forms. Science.

[15] Mitchell J.A., Warner T.D. (2006). COX isoforms in the cardiovascular system: Understanding the activities of non-steroidal anti-inflammatory drugs. Nat. Rev. Drug Discov..

[16] Branch G., Burgess D., Dunstan P., Foo L., Green G., Mack J.G., Ritchie E., Taylor W. (1972). Constituents of *Alphitonia* species. III. Alphitexolide, a new triterpene, and other extractives. Aust. J. Chem..

[17] Setzer W.N., Petty J.L., Schmidt J.M., Setzer M.C., Bates R.B., Jackes B.R. (2004). Bioactive lupane triterpenoids in *Alphitonia petriei* from Paluma, North Queensland, Australia. Curr. Top. Phytochem..

[18] Julian P.L., Pikl J., Dawson R. (1938). The constituents of ceanothus americanus. I. Ceanothic acid. J. Am. Chem. Soc..

[19] Boyer J.P., Eade R.A., Locksley H., Simes J.J.H. (1958). Extractives of Australian timbers. II. Emmolic acid, a new triterpene acid from Emmenospermum alphitonoides F. Muell. Aust. J. Chem..

[20] Yagi A., Okamura N., Haraguchi Y., Noda K., Nishioka I. (1978). Studies on the constituents of *Zizyphi fructus* II. Structures of new *p*-coumaroylates of alphitolic acid. Chem. Pharm. Bull..

[21] Plastina P., Bonofiglio D., Vizza D., Fazio A., Rovito D., Giordano C., Barone I., Catalano S., Gabriele B. (2012). Identification of bioactive constituents of *Ziziphus jujube* fruit extracts exerting antiproliferative and apoptotic effects in human breast cancer cells. J. Ethnopharmacol..

[22] Moghaddam M.G., Ahmad F.B.H., Samzadeh-Kermani A. (2012). Biological activity of betulinic acid: A review. Pharmacol. Pharm..

[23] Hao J., Zhang X., Zhang P., Liu J., Zhang L., Sun H. (2009). Efficient access to isomeric 2,3-dihydroxy lupanes: First synthesis of alphitolic acid. Tetrahedron.

[24] Jeong W., Hong S.S., Kim N., Yang Y.T., Shin Y.S., Lee C., Hwang B.Y., Lee D. (2009). Bioactive triterpenoids from *Callistemon lanceolatus*. Arch. Pharm. Res..

[25] Aguirre M.C., Delporte C., Backhouse N., Erazo S., Letelier M.E., Cassels B.K., Silva X., Alegria S., Negrete R. (2006). Topical anti-inflammatory activity of 2alpha-hydroxy pentacyclic triterpene acids from the leaves of Ugni molinae. Bioorg. Med. Chem..

[26] Bai L.-Y., Chiu C.-F., Chiu S.-J., Chen Y.-W., Hu J.-L., Wu C.-Y., Weng J.-R. (2015). Alphitolic acid, an anti-inflammatory triterpene, induces apoptosis and autophagy in oral squamous cell carcinoma cells, in part, through a p53-dependent pathway. J. Funct. Foods.

