# Supplementary material for: Anti-Inflammatory Chemical Profiling of the Australian Rainforest Tree Alphitonia petriei (Rhamnaceae)
Source: Molecules. 2016 Nov 11;21(11):1521. doi: 10.3390/molecules21111521 (PMC6273140; doi:10.3390/molecules21111521)
Supplement: Supplementary file 1 [file molecules-21-01521-s001.pdf]

Supplementary Materials: Anti-Inflammatory Chemical Profiling of the Australian Rainforest Tree *Alphitonia petriei* (Rhamnaceae)

Ritesh Raju, Dhanushka Gunawardena, Most Afia Ahktar, Mitchell Low, Paul Reddell and Gerald Münch

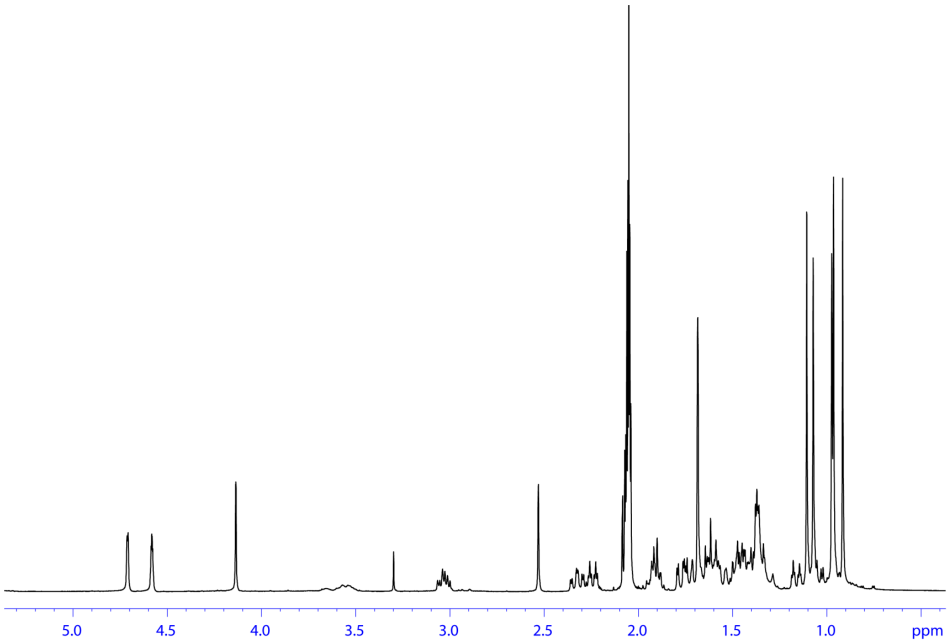

Figure S1. ^{1}H-NMR (400 MHz, Acetone-d_6) spectrum of emmolic acid (**1**).

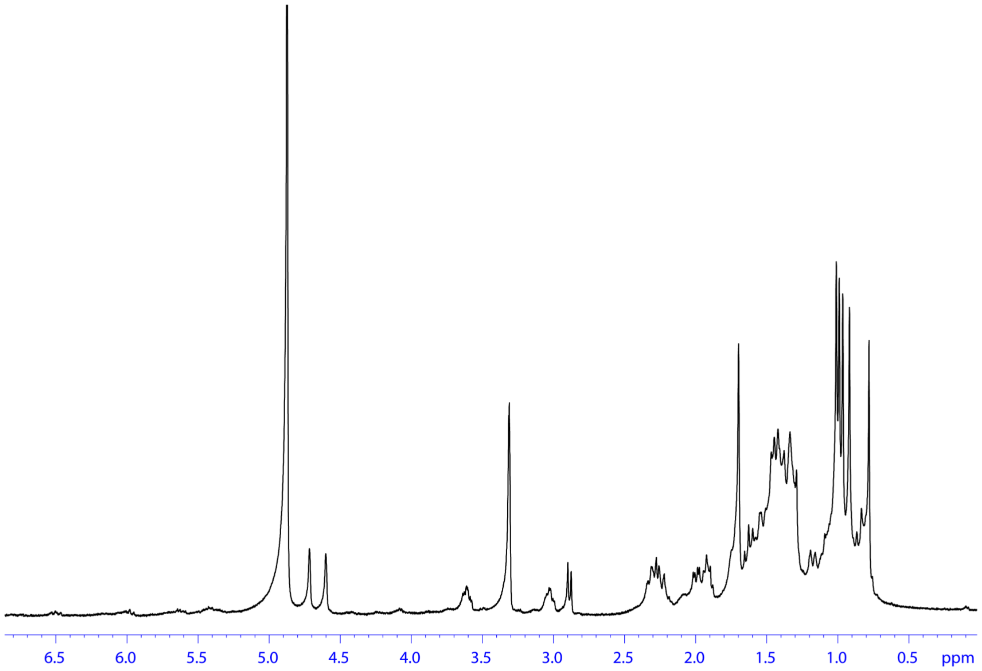

Figure S2. ^{1}H-NMR (400 MHz, Methanol-d_4) spectrum of alphitolic acid (**2**).

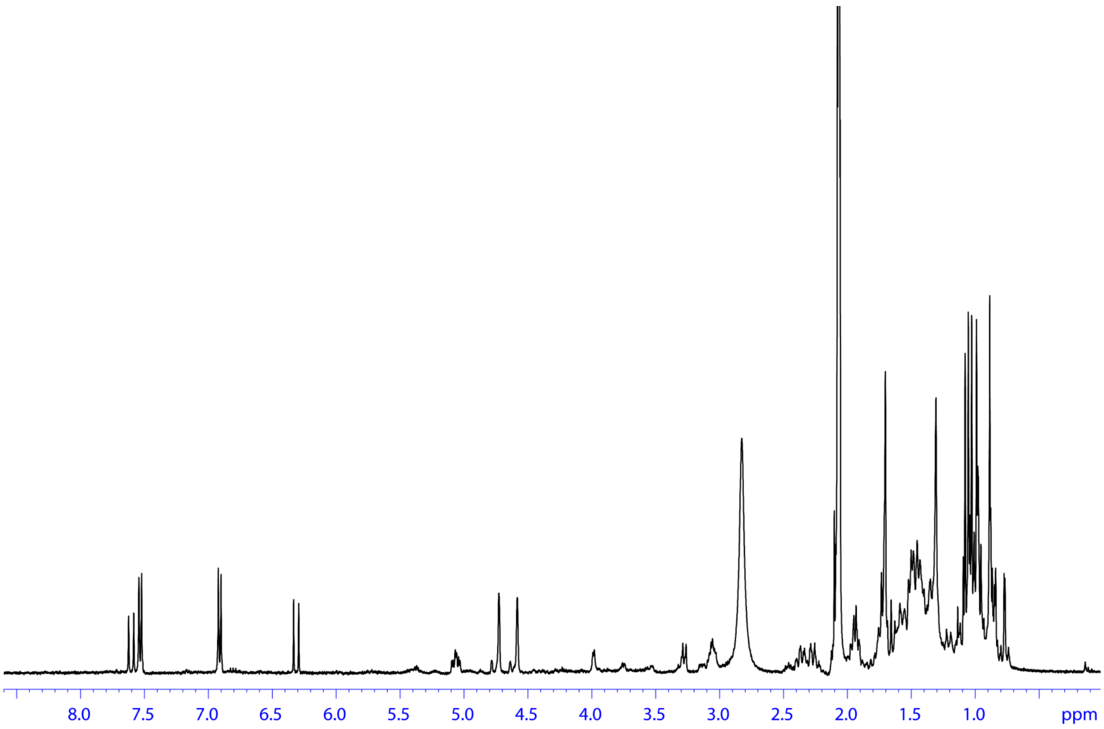

Figure S3. ^{1}H-NMR (400 MHz, Acetone-d_6) spectrum of *trans*-coumaroyl alphitolic acid (**3**).

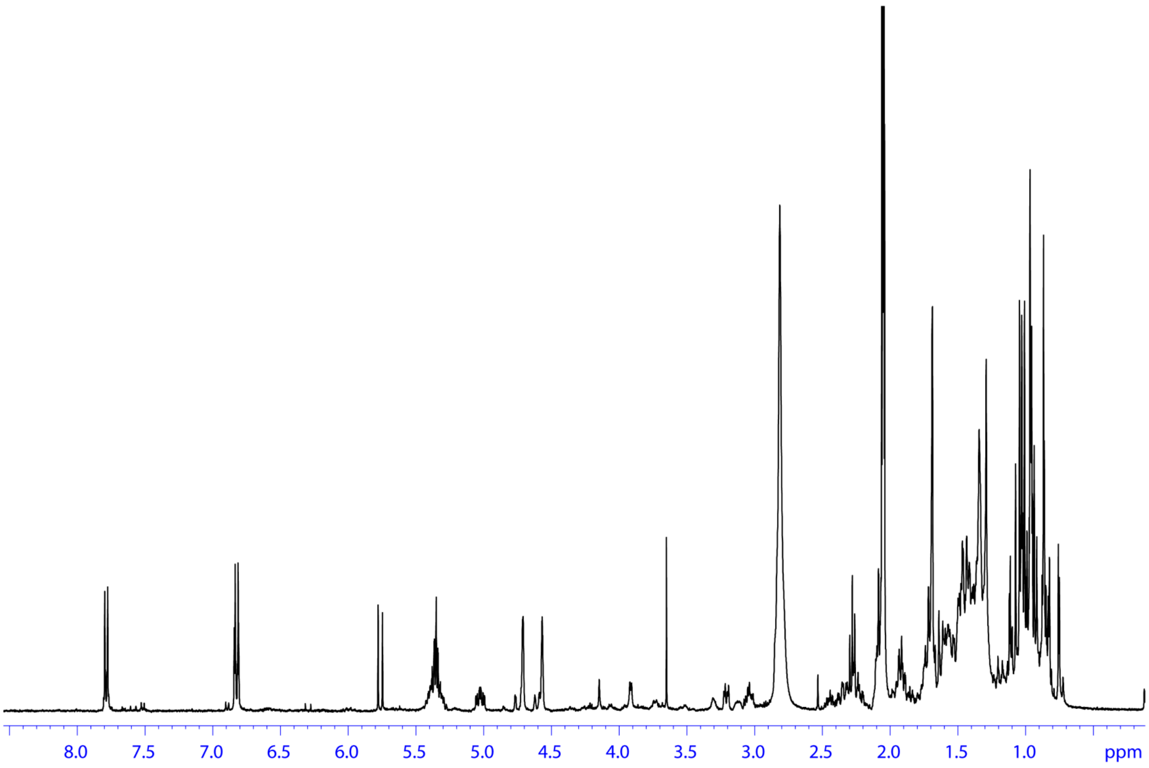

Figure S4. ^{1}H-NMR (400 MHz, Acetone-d_6) spectrum of *cis*-coumaroyl alphitolic acid (**4**).

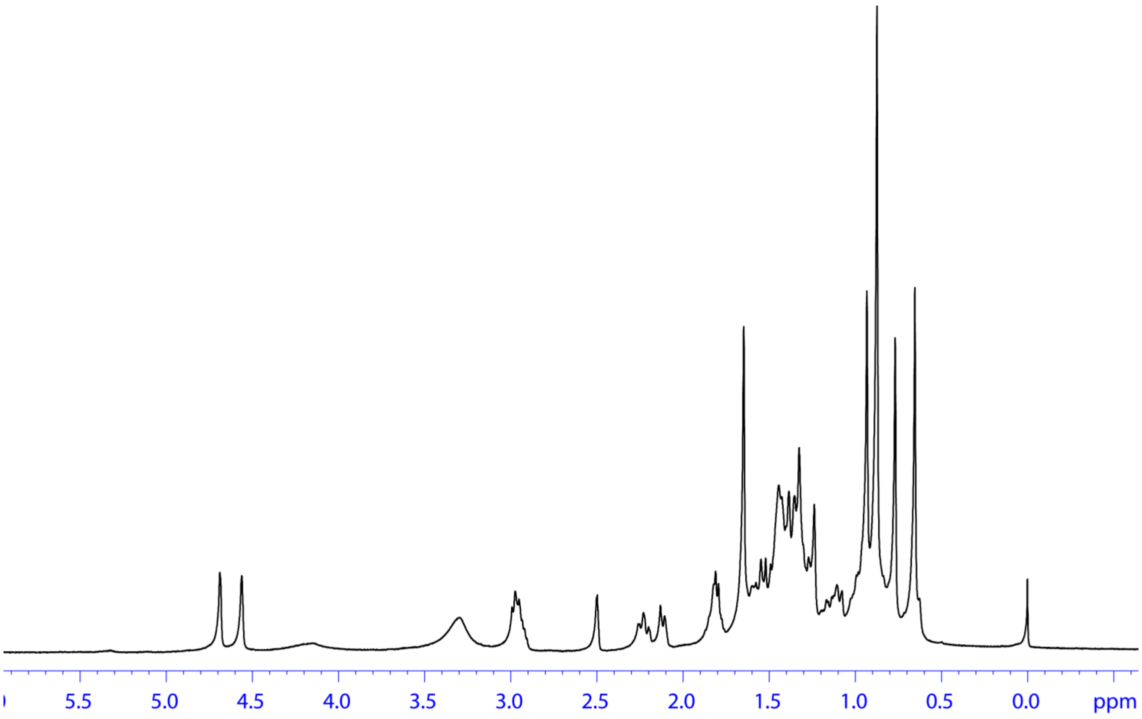

Figure S5. ^{1}H-NMR (400 MHz, DMSO-d_6) spectrum of betulinic acid (**5**).

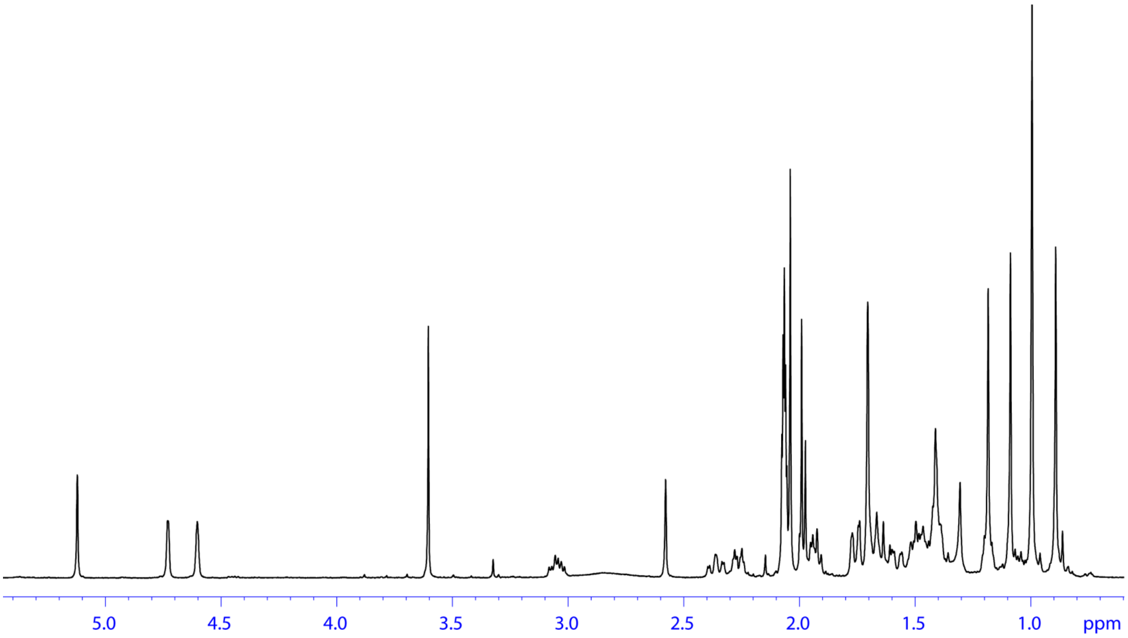

Figure S6. ^{1}H-NMR (400 MHz, Acetone-d_6) spectrum of emmolic acid acetate (**6**).

Table S1.-NMR (400 MHz, Acetone-d_6) data for (**1**).

**Pos**	**δ_H, Mult (*J* in Hz)**	**δ_C ***	**COSY**	**HMBC**
1	2.30, s	65.9		2, 3, 5, 9, 10, 25
2		176.7		
3	3.91, s	83.6		2, 4, 23
4		48.9		
5	1.59, m	56.3	6	
6	1.35, 1.47, m	23.3	5, 7	
7	1.30, m	34.0	6	
8		41.6		
9	1.52, m	48.8	11	
10		43.1		
11	1.55, 0.92, m	25.5	9, 12	
12	1.46, m	23.5	11, 13	
13	2.20, m	38.2	12, 18	
14		42.9		
15	1.42, 1.10, m	29.9	16	
16	1.80, 1.33, m	30.3	15	28
17				
18	1.53, m	48.9	13, 19	28
19	3.03, m	47.0	18, 21	
20		150.6		
21	1.81, 1.42, m	36.7	22	
22	1.36, m	38.8	21	
23	1.07, s	30.3		3, 4, 5, 24
24	0.91 s	18.7		4, 5, 23
25	1.11, s	17.8		1, 9, 10
26	0.96, s	16.2		8, 9, 14
27	0.97, s	14.2		8, 13, 14, 15
28		177.6		
29	4.71, s, 4.58, s	110.1		19, 30
30	1.69, s	19.4		19, 20, 29

* assignments supported by HSQC and HMBC experiments.

Table S2.-NMR (400 MHz, MeOH-d_4) data for (**2**).

Pos	δ_H, Mult (*J* in Hz)	δ_C *	COSY	HMBC
1	1.99, 0.84, m	48.3	2	2, 3, 5, 10
2	3.62, m	69.7	1, 3	
3	2.89, d (*9.4*)	84.3	2	2, 4, 23, 24
4		40.5		
5	0.81, m	56.7	6	
6	1.45, m [a]	19.1	5, 7	
7	1.42, m [a]	35.3	6	
8		42.0		
9	1.37, m	52.0	11	
10		39.4		
11	1.46, 1.31, m	22.0	9, 12	
12	1.70, 1.10, m	26.7	11, 13, 18	
13	2.31, m	39.5	12	
14		43.4		
15	1.20	30.8	16	
16	2.24, 1.42, m	33.2	15	
17		57.4		
18	1.63, m	50.3	13, 19	
19	3.02, m	48.4	18, 21	
20		151.8		
21	1.95, 1.40, m	31.6	19, 22	
22	2.28, m	34.7	21	
23	0.99, s	29.1		3, 4, 5, 24
24	0.78, s	17.1		3, 4, 5, 23
25	0.92, s	17.8		1, 5, 9, 10
26	0.97, s	16.5		7, 8, 9, 14
27	1.01, s	14.9		8, 13, 14, 15
28		179.7		
29	4.72, 4.60, s	110.2		19, 30
30	1.70, s	19.4		19, 20, 29

* assignments supported by HSQC and HMBC experiments; [a] overlapping signals.

Table S3.-NMR (400 MHz, Acetone-d_6) data for (**3**).

Pos	δ_H, Mult (*J* in Hz)	δ_C *	COSY	HMBC
1	1.92, 0.90, m	40.2	2	
2	4.88, ddd (*11.2*, *10.0*, *4.4*)	72.6	1, 3	1′
3	3.10, dd (*10.0*, *5.7*)	78.6	2	1, 2, 23, 24
4		44.5		
5	0.83, m	55.0	6	
6	1.32 [b], 1.21, m	21.1	5, 7	
7	1.35 [c], m	34.1	6	
8		41.4		
9	1.51 [a], m	48.9	11	
10		43.0		
11	1.60, 0.98, m	25.3	9, 12	
12	1.50 [a], m	24.8	11, 13	
13	2.22, m	37.8	12, 18	
14		42.8		
15	1.42, 1.10, m	29.6	16	
16	1.80 [d], 1.31 [b], m	30.4	15	
17		57.3		
18	1.35 [c], m	50.0	13, 19	
19	2.94, m	47.0	18, 21	
20		150.6		
21	1.81 [d], 1.42, m	36.6	22	
22	1.34, m	34.0	21	
23	0.95, s	29.0		
24	0.74, s	17.2		
25	0.94, s	14.7		
26	0.87, s	16.0		
27	0.90, s	17.3		
28		177.6		
29	4.68, s, 4.54, s	110.1		
30	1.64, s	19.3		
1′		166.8		
2′	7.52 [e]	144.5		1′
3′	6.31, d (*16.0*)	115.5		1′, 4′
4′		125.6		
5′	7.51 [c], d (*8.3*)	130.5		3′, 7′, 9′
6′	6.80, d (*8.3*)	116.3		4′, 7′, 8′
7′		160.0		
8′	6.80, d (*8.3*)	116.3		4′, 6′, 7′
9′	7.51 [c], d (*8.3*)	130.5		3′, 5′, 7′

* assignments supported by HSQC and HMBC experiments; [a–e] overlapping signals.

Table S4.-NMR (400 MHz, Acetone-d_6) data for (**4**).

Pos	δ_H, Mult (*J* in Hz)	δ_C *	COSY	HMBC
1	1.92, 0.90, m	40.4	2	
2	4.88, ddd (*11.1*, *10.0*, *4.2*)	72.5	1, 3	1′
3	3.12, dd (*10.0*, *5.7*)	78.9	2	1, 2, 23, 24
4		44.0		
5	0.83, m	54.0	6	
6	1.32 [b], 1.21, m	21.0	5, 7	
7	1.35 [c], m	34.1	6	
8		41.2		
9	1.51 [a], m	48.7	11	
10		43.0		
11	1.60, 0.98, m	25.3	9, 12	
12	1.50 [a], m	24.6	11, 13	
13	2.23, m	37.8	12, 18	
14		42.8		
15	1.42, 1.10, m	29.6	16	
16	1.80 [d], 1.31 [b], m	30.4	15	
17		57.2		
18	1.35 [c], m	50.0	13, 19	
19	2.90, m	47.0	18, 21	
20		150.5		
21	1.81 [d], 1.42, m	36.6	22	
22	1.34, m	34.0	21	
23	0.93, s	28.0		
24	0.78, s	17.1		
25	0.96, s	14.4		
26	0.87, s	15.9		
27	0.90, s	17.3		
28		177.2		
29	4.71, s, 4.57, s	110.1		
30	1.60, s	19.3		
1′		166.8		
2′	6.83 [c]	144.5		1′
3′	5.77, d (*12.9*)	115.5		1′, 4′
4′		125.6		
5′	7.79, d (*8.3*)	130.7		3′, 7′, 9′
6′	6.83 [e], d (*8.3*)	116.4		4′, 7′, 8′
7′		160.0		
8′	6.83 [e], d (*8.3*)	116.4		4′, 6′, 7′
9′	7.79, d (*8.3*)	130.7		3′, 5′, 7′

* assignments supported by HSQC and HMBC experiments; [a–e] overlapping signals.

Table S5.-NMR (400 MHz, DMSO-d_6) data for (**5**).

Pos	δ_H, Mult (*J* in Hz)	δ_C *	COSY
1	1.89, 0.95, m	48.3	2
2	2.25, 2.09, m	39.7	1, 3
3	2.97, m	77.3	2
4		39.7	
5	0.80, m	55.9	6
6	1.43, m [a]	18.9	5, 7
7	1.42, m [a]	36.0	6
8		41.7	
9	1.40, m	52.5	11
10		39.4	
11	1.40, 1.38, m	22.0	9, 12
12	1.65, 1.08, m	25.5	11, 13, 18
13	2.36, m	39.9	12
14		42.0	
15	1.20	30.8	16
16	2.24, 1.42, m	33.2	15
17		57.4	
18	1.63, m	50.3	13, 19
19	3.02, m	48.4	18, 21
20		151.8	
21	1.95, 1.40, m	31.6	19, 22
22	2.28, m	34.7	21
23	0.77, s	29.1	
24	0.66, s	17.0	
25	0.87, s	17.7	
26	0.87, s	16.1	
27	0.93, s	14.6	
28		179.5	
29	4.69, 4.57, s	110.0	
30	1.65, s	19.4	

* assignments supported by HSQC and HMBC experiments; [a] overlapping signals.

Table S6.-NMR (400 MHz, Acetone-*d*₆) data for (**6**).

Pos	δ_H, Mult (*J* in Hz)	δ_C *	COSY	HMBC
1	2.58, s	62.7		2, 3, 5, 9, 10, 25
2		174.3		
3	5.12, s	84.8		1, 2, 4, 23
4		49.2		
5	1.62, m	56.5	6	
6	1.33, 1.45, m	23.3	5, 7	
7	1.30, m	34.0	6	
8		41.6		
9	1.52, m	48.8	11	
10		42.9		
11	1.55, 0.92, m	25.5	9, 12	
12	1.46, m	23.4	11, 13	
13	2.19, m	38.2	12, 18	
14		42.9		
15	1.42, 1.10, m	29.9	16	
16	1.80, 1.33, m	30.5	15	28
17				
18	1.53, m	48.9	13, 19	28
19	2.94, m	47.0	18, 21	
20		150.5		
21	1.81, 1.42, m	36.7	22	
22	1.36, m	38.8	21	
23	1.20, s	29.9		3, 4, 5, 24
24	1.10, s	17.5		4, 5, 23
25	0.88, s	19.1		1, 9, 10
26	1.00, s	16.5		8, 9, 14
27	0.99, s	14.9		8, 13, 14, 15
28		177.6		
29	4.71, s, 4.58, s	110.1		19, 30
30	1.64, s	19.4		19, 20, 29
31	2.04, s	19.9		32
32		169.6		

* assignments supported by HSQC and HMBC experiments.

Table S7. Statistical analysis of differences in IC_{50} values of compounds **1**–**6** and the control NSAIDs derived from Table 1 using the Student-Newman-Keuls Multiple Comparison Test.

Compaired Pair	Significant? $p < 0.05$?	Summary
**Nitric Oxide (*n* = 8)**		
**3** vs. Ibuprofen	Yes	***
**3** vs. Diclophenac	Yes	***
**3** vs. **1**	No	ns
**3** vs. **2**	No	ns
**3** vs. **6**	No	ns
**3** vs. **5**	No	ns
**3** vs. **4**	No	ns
**4** vs. Ibuprofen	Yes	***
**4** vs. Diclophenac	Yes	***
**4** vs. **1**	No	ns
**4** vs. **2**	No	ns
**4** vs. **6**	No	ns
**4** vs. **5**	No	ns
**5** vs. Ibuprofen	Yes	***
**5** vs. Diclophenac	Yes	***
**5** vs. **1**	No	ns
**5** vs. **2**	No	ns
**5** vs. **6**	No	ns
**6** vs. Ibuprofen	Yes	***
**6** vs. Diclophenac	Yes	***
**6** vs. **1**	No	ns
**6** vs. **2**	No	ns
**2** vs. Ibuprofen	Yes	***
**2** vs. Diclophenac	Yes	***
**2** vs. **1**	No	ns
**1** vs. Ibuprofen	No	ns
**1** vs. Diclophenac	No	ns
Diclophenac vs. Ibuprofen	Yes	***
**TNF-α (*n* = 3)**		
**4** vs. Ibuprofen	Yes	***
**4** vs. Diclophenac	No	ns
**4** vs. **6**	No	ns
**4** vs. **1**	No	ns
**4** vs. **5**	No	ns
**4** vs. 2	No	ns
**4** vs. **3**	No	ns
**3** vs. Ibuprofen	Yes	***
**3** vs. Diclophenac	No	ns
**3** vs. **6**	No	ns
**3** vs. **1**	No	ns
**3** vs. **5**	No	ns
**3** vs. **2**	No	ns
**2** vs. Ibuprofen	Yes	***
**2** vs. Diclophenac	No	ns
**2** vs. **6**	No	ns
**2** vs. **1**	No	ns
**2** vs. **5**	No	ns
**5** vs. Ibuprofen	Yes	***
**5** vs. Diclophenac	No	ns
**5** vs. **6**	No	ns
**5** vs. **1**	No	ns
**1** vs. Ibuprofen	No	ns
**1** vs. Diclophenac	No	ns
**1** vs. **6**	No	ns
**6** vs. Ibuprofen	No	ns
**6** vs. Diclophenac	No	ns
Diclophenac vs. Ibuprofen	No	ns

Single Mass Analysis
Tolerance = 100.0 PPM / DBE: min = 0.0, max = 50.0
Element prediction: Off
Number of isotope peaks used for i-FIT = 3

Monoisotopic Mass, Odd and Even Electron Ions
1 formula(e) evaluated with 1 results within limits (all results (up to 1000) for each mass)
Elements Used:
C: 28-32 H: 40-50 O: 0-5
3003fr2 in MeOH
20141201_3003fr2 114 (2.029) Cm (111:121) 1: TOF MS ES+
5.26e+004

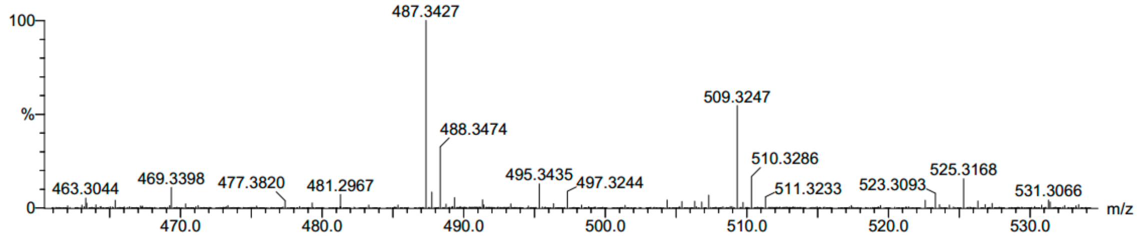

Mass	Calc. Mass	mDa	PPM	DBE	i-FIT	i-FIT (Norm)	Formula
Minimum:				0.0			
Maximum:		5.0	100.0	50.0			
487.3427	487.3424	0.3	0.6	7.5	257.4	0.0	C30 H47 O5

Figure S7. HRMS spectrum for emmolic acid (**1**).

Tolerance = 100.0 PPM / DBE: min = 0.0, max = 50.0
Element prediction: Off
Number of isotope peaks used for i-FIT = 3

Monoisotopic Mass, Even Electron Ions
3 formula(e) evaluated with 2 results within limits (all results (up to 1000) for each mass)
Elements Used:
C: 28-32 H: 40-50 O: 0-5
3003fr5 in MeOH
20141201_3003fr5 51 (0.914) Cm (48:51) 1: TOF MS ES+
4.33e+002

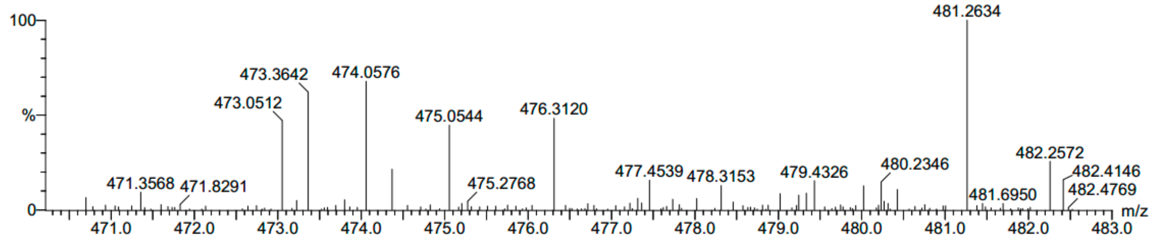

Mass	Calc. Mass	mDa	PPM	DBE	i-FIT	i-FIT (Norm)	Formula
Minimum:				0.0			
Maximum:		5.0	100.0	50.0			
473.3642	473.3631	1.1	2.3	6.5	121.7	0.6	C30 H49 O4

Figure S8. HRMS spectrum for alphitolic acid (**2**).

Single Mass Analysis
Tolerance = 100.0 PPM / DBE: min = 0.0, max = 50.0
Element prediction: Off
Number of isotope peaks used for i-FIT = 3

Monoisotopic Mass, Even Electron Ions
1 formula(e) evaluated with 1 results within limits (all results (up to 1000) for each mass)
Elements Used:
C: 37-40 H: 52-56 O: 4-7 Na: 0-1
3003fr8 in MeOH
20141201_3003fr8 162 (2.862) Cm (160:169)

1: TOF MS ES+
1.86e+004

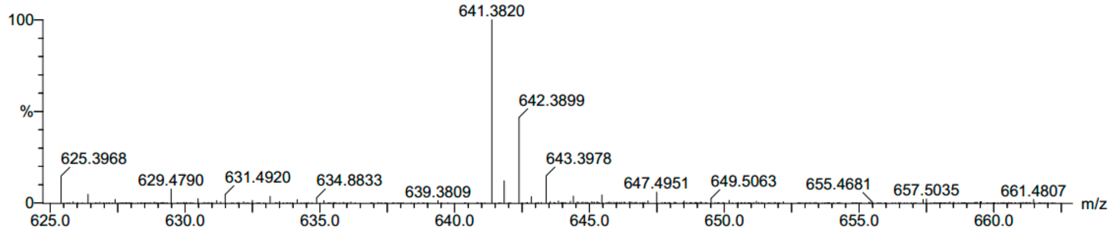

Minimum:				0.0			
Maximum:		5.0	100.0	50.0			
Mass	Calc. Mass	mDa	PPM	DBE	i-FIT	i-FIT (Norm)	Formula
641.3820	641.3818	0.2	0.3	12.5	121.5	0.0	C39 H54 O6 Na

Figure S9. HRMS spectrum for *trans*-coumaroyl alphitolic acid (**3**).

Single Mass Analysis
Tolerance = 100.0 PPM / DBE: min = 0.0, max = 50.0
Element prediction: Off
Number of isotope peaks used for i-FIT = 3

Monoisotopic Mass, Even Electron Ions
1 formula(e) evaluated with 1 results within limits (all results (up to 1000) for each mass)
Elements Used:
C: 37-40 H: 52-56 O: 4-7 Na: 0-1
3003fr9 in MeOH
20141201_3003fr9 74 (1.320) Cm (71:76)

1: TOF MS ES+
5.03e+003

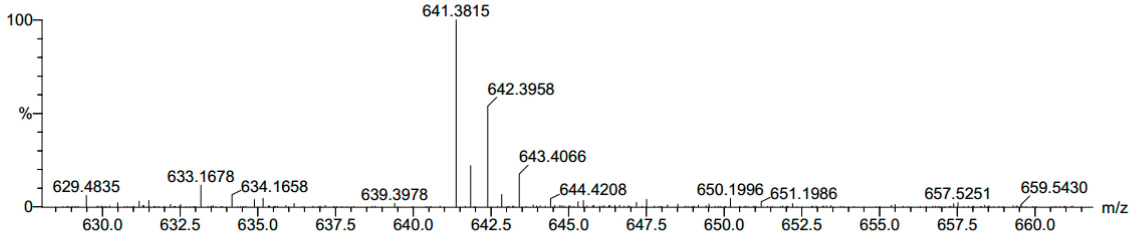

Minimum:				0.0			
Maximum:		5.0	100.0	50.0			
Mass	Calc. Mass	mDa	PPM	DBE	i-FIT	i-FIT (Norm)	Formula
641.3815	641.3818	-0.3	-0.5	12.5	85.4	0.0	C39 H54 O6 Na

Figure S10. HRMS spectrum for *cis*-coumaroyl alphitolic acid (**4**).

Single Mass Analysis
Tolerance = 100.0 PPM / DBE: min = 0.0, max = 50.0
Element prediction: Off
Number of isotope peaks used for i-FIT = 3

Monoisotopic Mass, Even Electron Ions
6 formula(e) evaluated with 4 results within limits (all results (up to 1000) for each mass)
Elements Used:
C: 28-32 H: 40-50 O: 0-5 Na: 0-1
3003fr10 in MeOH
20141201_3003fr10 121 (2.149) Cm (114:131)

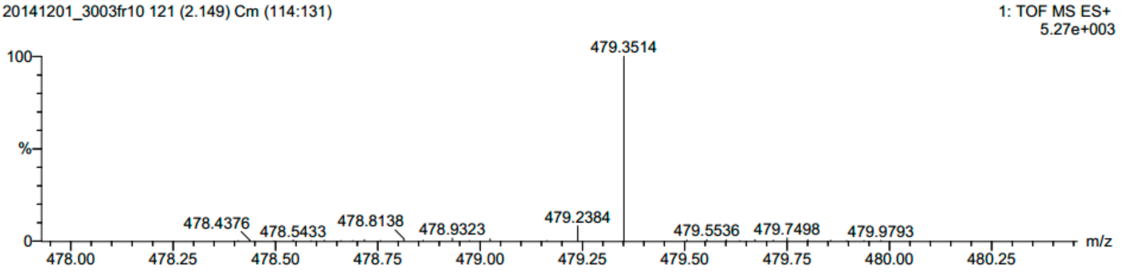

Minimum:				0.0			
Maximum:		5.0	100.0	50.0			
Mass	Calc. Mass	mDa	PPM	DBE	i-FIT	i-FIT (Norm)	Formula
479.3514	479.3501	1.3	2.7	6.5	98.3	0.9	C30 H48 O3 Na

Figure S11. HRMS spectrum for betulinic acid (**5**).

Single Mass Analysis
Tolerance = 50.0 mDa / DBE: min = -1.5, max = 50.0
Element prediction: Off
Number of isotope peaks used for i-FIT = 3

Monoisotopic Mass, Even Electron Ions
8 formula(e) evaluated with 1 results within limits (up to 50 closest results for each mass)
Elements Used:
C: 30-32 H: 47-48 O: 0-8 Na: 0-2
20160907_Ritesh_alphiacetate 96 (1.701)
1: TOF MS ES+

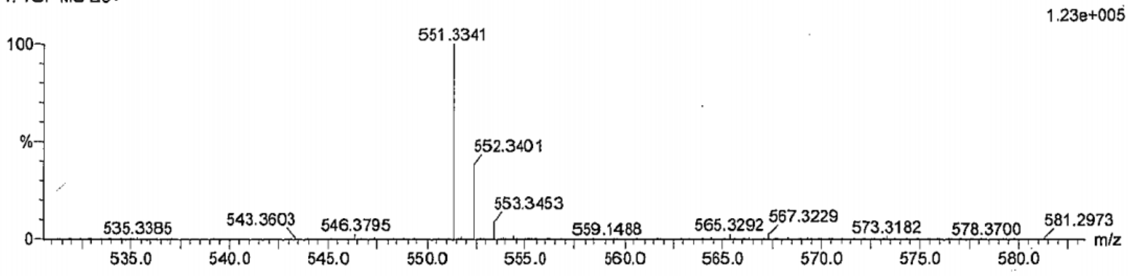

Minimum:				-1.5			
Maximum:		50.0	15.0	50.0			
Mass	Calc. Mass	mDa	PPM	DBE	i-FIT	i-FIT (Norm)	Formula
551.3341	551.3349	-0.8	-1.5	8.5	223.3	0.0	C32 H48 O6 Na

Figure S12. HRMS spectrum for emmolic acid acetate (**6**).
